# Risk of Colonization with Multidrug-Resistant Gram-Negative Bacteria Among Travellers and Migrants: A Narrative Review

**DOI:** 10.3390/tropicalmed10010026

**Published:** 2025-01-18

**Authors:** Diogo Mendes Pedro, Daniela Santos, Maria Meneses, Fátima Gonçalves, Gonçalo Jantarada Domingos, Cátia Caneiras

**Affiliations:** 1 Laboratory of Microbiology Research in Environmental Health (EnviHealthMicro Lab), Institute of Environmental Health (ISAMB), Associate Laboratory TERRA, Faculdade de Medicina, Universidade de Lisboa, 1249-028 Lisboa, Portugal; danielaaasantos@outlook.com (D.S.); mffmeneses@gmail.com (M.M.); ccaneiras@medicina.ulisboa.pt (C.C.); 2 Infectious Diseases Department, ULS Santa Maria, 1649-028 Lisboa, Portugal; fatima.s.goncalves@ulssm.min-saude.pt (F.G.); goncalo.domingos@ulssm.min-saude.pt (G.J.D.); 3 Infectious Diseases University Clinic, Faculdade de Medicina, Universidade de Lisboa, 1249-028 Lisboa, Portugal; 4 Institute of Pharmacology and Neurosciences, Faculdade de Medicina, Universidade de Lisboa, 1249-028 Lisboa, Portugal; 5 Egas Moniz Interdisciplinary Research Center, Egas Moniz School of Health & Science, Monte da Caparica, 2829-511 Almada, Portugal; 6 Institute of Preventive Medicine and Public Health (IMP&SP), Faculdade de Medicina, Universidade de Lisboa, 1249-028 Lisboa, Portugal

**Keywords:** Enterobacterales, *Pseudomonas aeruginosa*, travel medicine, travel-related bacterial colonization, antimicrobial resistance, ESBL, carbapenem resistance, epidemiology, global health

## Abstract

Globalization in the 21st century has posed several challenges. In particular, the spread of multidrug-resistant bacterial strains, especially Gram-negative bacteria, which are prevalent in certain regions of the world, is one of the most critical issues. This raises concerns about the risks associated with the booming tourism industry and migratory flows. In fact, even transient colonization with multidrug-resistant strains can present significant challenges to individual, family, and public health. Understanding the epidemiology and mechanisms of resistance, associated risk factors and prevention policies is therefore essential to ensure that strategies are in place to limit the global spread of high-risk bacterial clones and thereby protect public health.

## 1. Introduction

Globalization and the increasing ease of international travel in the 21st century for tourism, migration, and warfare have led to significant health concerns, particularly regarding the spread of multidrug-resistant (MDR) pathogens. With over a billion international trips taken annually [1], the movement of people has become a key factor in the transmission of antimicrobial-resistant (AMR) bacteria. While travellers or migrants may not always experience clinical infection from these pathogens, colonization with MDR bacteria can occur, acting as a silent reservoir for further transmission and potential infection, especially in individuals with underlying health conditions or those undergoing medical procedures abroad [2,3].

MDR bacteria, which exhibit acquired non-susceptibility to at least three antimicrobial classes, present a growing public health challenge worldwide. These include extended-spectrum β-lactamase (ESBL)-producing Enterobacterales, AmpC β-lactamase-producing Enterobacterales, carbapenem-resistant Enterobacterales (CRE), *Pseudomonas aeruginosa* with difficult-to-treat resistance, carbapenem-resistant *Acinetobacter baumannii*, and *Stenotrophomonas maltophilia*. These bacteria can colonize different parts of the body, such as the gastrointestinal tract, respiratory system, and skin, without necessarily causing disease. However, such colonization can serve as a precursor to infection, particularly in immunocompromised individuals. The risk of colonization with MDR organisms is amplified among international travellers or migrants, particularly in regions with high AMR rates and suboptimal infection control measures [4]. In this context, understanding the mechanisms of resistance, the risk factors for colonization, and the strategies to mitigate the spread of MDR bacteria is critical.

The concern regarding colonization by MDR strains in travellers is not recent. In the 1990s, before the spread of ESBL-producing Enterobacterales in the community was observed, Murray et al. [5] had already noted that American travellers to Mexico could acquire MDR *Escherichia coli* (documenting resistance to ampicillin, chloramphenicol, trimethoprim-sulfamethoxazole, tetracycline, and/or streptomycin) during their travels. At that time, there was little concern, as susceptibility to several drugs, including third-generation cephalosporins—novel drugs at the time—was still observed.

This article aims to provide a review of the risk of MDR colonization among travellers and migrants, with a specific focus on Enterobacterales and *P. aeruginosa*, the most critical Gram-negative bacteria when considering AMR in Europe. The authors will explore the mechanisms of resistance, factors that contribute to MDR acquisition, and preventive measures to reduce the risk of colonization with these problematic pathogens.

## 2. Defining MDR Bacteria

Multidrug-resistant (MDR) bacteria are those that have developed resistance to multiple classes of antibiotics, making infections caused by these organisms more challenging to treat with standard antimicrobial therapies. Resistance can develop through genetic mutations, horizontal transfer of resistance genes, or selective pressure from the overuse or misuse of antibiotics. In 2014, the Review on Antimicrobial Resistance projected that 10 million deaths caused by AMR could occur by 2050, with an associated cost of 100 trillion USD in global economic output [6]. In 2015, an estimated 33,110 people died due to AMR in Europe, while in Portugal the number was estimated to be 1158 [7]. The World Health Organization (WHO) has highlighted AMR as one of the most critical threats to global health in the 21st century. In 2019, bacterial AMR was directly responsible for 1.27 million deaths worldwide and contributed to 4.95 million deaths [8]. Although the use of antibiotics itself is the primary determinant of antimicrobial resistance [9], it is also linked to poor infection prevention and control practices, particularly in healthcare settings [10].

MDR bacteria can be classified into several categories, including those resistant to beta-lactams, aminoglycosides, fluoroquinolones, and carbapenems, among others. The most widely accepted definition of multidrug resistance consists of acquired non-susceptibility to at least one agent from three or more antimicrobial classes [11]. The consequences of MDR bacteria include prolonged hospital stays, more expensive treatments, and higher mortality rates, with some infections becoming essentially untreatable with existing antibiotics [12,13,14].

The primary groups of MDR bacteria that are of particular concern in the context of international travel include Enterobacterales (primarily ESBL-producing *E. coli* or *Klebsiella pneumoniae*), *P. aeruginosa*, *Acinetobacter baumannii*, and *Staphylococcus aureus*. These organisms are often implicated in serious healthcare-associated infections, including urinary tract infections, bloodstream infections, and pneumonia [2].

The rise of AMR is a direct result of the use of antimicrobial agents. In clinical practice, the indiscriminate use of antimicrobials in the treatment of infectious diseases, both in the community and in hospital settings, facilitates the elimination of susceptible strains and recolonization by resistant strains. Selective pressure can also be observed, not through the elimination of the susceptible bacteria, but through the selection of bacteria possessing inducible resistance genes. The antimicrobial can induce the expression of a resistance gene, just as it may induce a mutation in the resistance gene [10].

The emergence, evolution, and spread of resistance can be attributed to several factors, including misdiagnosis of infectious diseases, patient pressure, and the influence of advertising, as well as a lack of educational programmes and adequate information. Indeed, non-compliance with prescribed therapy or self-medication (especially in countries where antimicrobials are available over the counter [15,16]) and the ’poverty paradigm’ [12], which involves limited access to drugs, under-dosing, and the use of counterfeit drugs (especially in developing countries) further exacerbate the problem. The inappropriate use of antibiotics in hospital settings, the overuse of empirical therapy, the resistance to the de-escalation of antimicrobials and their use in agriculture, aquaculture, and veterinary medicine are also critical factors [17,18,19]. The widespread use of antimicrobial products such as disinfectants and antiseptics in household items, the lack of potable water, inadequate sanitary facilities, limited access to proper hygiene, and globalization, which increases opportunities for international travel and trade, also contribute to the issue [12,13,20].

The key consequences of microbial resistance include increased mortality, as empirical therapies are bound to fail in often severe infections; increased morbidity, resulting in prolonged illnesses, frequent hospitalizations, and a higher likelihood of spreading resistant microorganisms; higher costs due to the use of newer, more expensive drugs and extended hospital stays; and limited solutions, with fewer new drugs being developed and designed [12].

## 3. Mechanisms of Resistance in Gram-Negative Bacteria

There are several resistance mechanisms in Gram-negative bacteria. These render commonly used antibiotics potentially ineffective, leading to limited treatment options [21]:

- Production of antibiotic-inactivating enzymes: Production of enzymes able to inactivate antibiotics is the most frequent mechanism of beta-lactams resistance among Gram-negative bacteria. ESBL can hydrolyse extended-spectrum beta-lactam antibiotics (such as third-generation cephalosporins) and other beta-lactams, such as penicillins. ESBL-producing strains of *E. coli* and *Klebsiella pneumoniae* have become endemic in many parts of the world, posing a significant threat to public health. However, these beta-lactamases are unable to degrade carbapenems. *P. aeruginosa* also produces a variety of beta-lactamases, including cephalosporinases and carbapenemases, which degrade beta-lactam antibiotics. The emergence of CRE, driven by carbapenemase production, is a major cause for concern. These enzymes, including KPC (*Klebsiella pneumoniae* carbapenemase) and OXA-48, can degrade carbapenems and nearly all other beta-lactams, leaving mostly only combinations with the latest carbapenemase inhibitors as viable therapeutic options among beta-lactams. A subset of these enzymes, metallo-beta-lactamases (such as the New Delhi metallo-beta-lactamase—NDM), are especially nefarious, given that they are not inhibited by any beta-lactamase inhibitors. As aztreonam is not degraded by these enzymes, it could be an interesting therapeutic alternative. However, it is not uncommon to observe the co-production of metallo-beta-lactamase and other enzymes (e.g., ESBL), especially in *P. aeruginosa*, in various regions of the world, rendering aztreonam ineffective by itself. Aminoglycoside-modifying enzymes are a key mechanism by which Gram-negative bacteria become MDR through antibiotic modification. They are crucial enzymes that catalyse the chemical modification of aminoglycoside antibiotics, resulting in their inactivity.
- Efflux pumps: Efflux pumps are transmembranar proteins that can actively export a variety of antibiotics from the cell, reducing their intracellular concentrations. They are responsible for the emergence of resistance to several antibiotic classes, such as tetracyclines, fluoroquinolones, aminoglycosides, and penicillins.
- Modifications of antibiotic targets: Modifications in drug target sites are one of the primary mechanisms by which Gram-negative bacteria acquire resistance to multiple antibiotics. For example, alterations in the lipopolysaccharide (LPS) present in the outer cell membrane enhance bacterial stability by protecting it from external threats. On the other hand, the addition of positively charged sugars decreases the negative charge of Lipid A, reducing its ability to bind cationic antimicrobial peptides and weakening electrostatic interactions, which are essential for polymixins such as colistin. Another target that can be modified is the 16S ribosomal RNA. Its methylation has emerged as a new resistance mechanism, specifically against drugs acting at this site, including aminoglycosides. Changes in penicillin-binding proteins (PBPs) can also result in a reduced ability of beta-lactams to bind to their target site, thereby decreasing their activity.
- Decreased porins permeability: Another mechanism used by many Gram-negative bacteria involves limiting the influx of antibiotics into the cell, thereby preventing their action on the therapeutic target. Mutations in porins, transmembrane proteins essential for the entry of hydrophilic drugs, severely limit the efficacy of drugs such as beta-lactams, fluoroquinolones, and tetracyclines. Another important resistance mechanism is remodelling of the outer cell membrane, allowing the bacteria to regulate its membrane by removing or adding specific components (such as lipids or proteins), enabling adaptation to a new environment.
- Biofilm formation: Some bacteria, in particular *P. aeruginosa*, can form biofilms on medical devices and host tissues, which protect them from both the host immune response and antibiotic treatment. Biofilms are a major contributor to chronic infections, particularly in patients with cystic fibrosis and those with prosthetic devices.

In travellers, colonization by MDR Enterobacterales is common, especially by ESBL-producing *E. coli* (88–100% in some studies [22,23,24]). ESBL-producing *K. pneumoniae* is also occasionally found, along with other ESBL-producing Enterobacterales, including non-Typhi *Salmonella* species. Polymicrobial colonization has also been frequently observed [22]. The most encountered ESBLs are CTX-M enzymes, particularly group 1 (including CTX-M-15, the most frequent in Portugal) [22,25]. Colonization with AmpC plasmid-producing strains is less frequent [22,23,26].

Regarding the acquisition of CRE or MDR *P. aeruginosa*, this seems to be substantially less common. Although AMR with these enzymes is becoming more frequent in certain regions of the world, especially South Asia, there is little evidence in the literature on the acquisition of these strains, possibly due to study protocols mainly focusing on ESBL colonization [2]. This is particularly relevant for metallo-beta-lactamases, enzymes rarely found in Portugal and notable for being frequently imported from elsewhere [25,27,28]. Epidemiological studies suggest that intercontinental travel to endemic regions contributes significantly to the global dissemination of strains harbouring these mechanisms of resistance [29,30]. Nevertheless, infection with *P. aeruginosa* has been found, especially after contact with contaminated recreational waters, particularly in an outbreak setting [31,32]. Regarding MDR *P. aeruginosa* strains, these are mostly associated with the use of healthcare facilities, although colonization is quite rare when compared with Enterobacterales [2].

## 4. Epidemiology of International Travel and MDR Risk

The 21st century has been characterized by an exponential increase in the number of international travels, whether by air, land, or sea. Indeed, in 2019, prior to the COVID-19 pandemic, which naturally caused a decline in the annual number of trips, approximately 1.5 billion trips were recorded [33]. At that time, in Portugal, a country with over 10 million residents, there were around 24.6 million tourist arrivals and just over 3 million trips abroad [34]. In 2010, projections estimated nearly 2 billion annual trips by 2030, a target likely to be met (or even exceeded) despite the pandemic [35,36].

However, not all travels are for tourism purposes. There is a significant annual number of migrants and refugees, which has notably increased this decade due to ongoing armed conflicts and social instability. Indeed, human conflict is a well-established driver of AMR, not only due to a reduction in infection control priorities, potential damage to diagnostic, water, and sanitation infrastructure, and an increase in high-risk injuries, but also because of an increased migratory flow potentially carrying MDR strains [37]. The WHO estimates that 108.4 million people were forcibly displaced by the end of 2022 due to these reasons [38]. Specifically in Portugal, there are more than 750,000 immigrants, with nearly one-third of them coming from Brazil, around 15% from Africa (especially African Portuguese-speaking countries), and another 15% from Asia [39].

## 5. Regions with Higher Risk of MDR Strains

Certain countries and regions present a high risk of acquiring MDR bacterial infections. Factors such as misuse of antibiotics, inadequate infection control in healthcare settings, and poor sanitation contribute to the heightened risk [12]. Low- and middle-income tropical and subtropical countries in Southeast Asia, Sub-Saharan Africa, and parts of Latin America exhibit especially high levels of AMR [40].

India, in particular, demonstrates high rates of resistance to commonly used antibiotics, with 60–80% of Enterobacterales producing ESBL [41] and more than 60% fluoroquinolone resistance in *E. coli* [42]. Carbapenem resistance in *K. pneumoniae* has been reported in approximately 60% of isolates [43]. Regarding *P. aeruginosa* strains, molecular studies show the presence of carbapenemase-encoding genes in up to 60% of isolates, with metallo-beta-lactamases being the most prevalent [44].

In China, the prevalence of ESBL-producing Enterobacterales has been described as 37.2% in a large multicentre study [45]. Carbapenem-resistant *P. aeruginosa* varies depending on region, but has been reported as around 5–40% [46,47]. A similar trend is observed in Thailand, where strains of *E. coli* and *K. pneumoniae* produced ESBL in 42.5% and 30.2% of cases, with a high rate of concomitant resistance to fluoroquinolones and trimethoprim/sulfamethoxazole, but with significant regional variation [48,49,50,51]. Likewise, in Southeast Asian countries, the prevalence of ESBL-producing Enterobacterales varies significantly, with rates of 19.8% in Singapore, 36.8% in the Philippines, and 40.6% in Cambodia [52,53]. Regarding *P. aeruginosa*, resistance to carbapenems in Thailand and elsewhere in the Asia-Pacific Region is prevalent, with production of metallo-beta-lactamases being widely prevalent [54,55].

This trend continues among other low-income countries, particularly in Africa. For example, Egypt reports 75.6% MDR *P. aeruginosa*, not unlike some Middle-Eastern countries [56]. Data from sub-Saharan Africa is scarce; however, ESBL-producing Enterobacterales prevalence seems to range from 11 to 72%. Regarding carbapenemase-producing strains, the prevalence of isolates in hospital settings ranges from 2.3% to 67.7% in North Africa and from 9% to 60% in sub-Saharan Africa. As for the African Portuguese-speaking countries, there is evidence of metallo-beta-lactamase transmission, at least in Angola [57,58,59].

In Latin America, there is a 11–25% and 45–53% prevalence of *E. coli* and *K. pneumoniae* isolates, respectively, that are resistant to third-generation cephalosporins, depending on region [60]. In *P. aeruginosa* strains, the ATLAS programme reports 33.1% of meropenem-resistance prevalence [61].

## 6. Risk of MDR Gram-Negative Colonization in Travellers and Migrants

Although the first studies focusing on the transmission of multidrug-resistant bacteria in travellers date back to the 1990s [5], this concern gained traction, particularly in Northern European countries. In 2010, Tham et al., demonstrated that 24% of Swedish patients with gastrointestinal complaints after travelling had been colonized by ESBL-producing Enterobacterales. Notably, this was primarily observed in travellers to non-European countries (37%, especially if travelling to India—79%) compared to those travelling within Europe (3%) [4]. The risk factors are depicted in Table 1.

In another study, a German cohort found that while 3% of travellers were colonized before travelling to low- or middle-income tropical or subtropical countries, 23% were colonized by ESBL-producing Enterobacterales during their travels. A higher risk was observed, particularly in trips to Asia (especially Southern Asia), followed by Africa (mainly Eastern Africa) [62,63]. Other European cohorts show similar incidence rates [3,64,65]. Travelling to the Indian subcontinent appears to present the highest risk of MDR Gram-negative acquisition, with rates ranging from 20% to 69% (a 24.8-fold risk increase in MDR Gram-negative colonization versus 8.6-fold for the rest of Asia). A stay in the Middle East or North Africa (13% to 44%), in Sub-Saharan Africa (10% to 47%) or Latin America (0% to 31%) seems to have a lower risk, albeit with variations regarding specific regions and travel characteristics [40,66], likely reflecting the geographical variations in AMR epidemiology, due to the economic, social, and public health differences in each region.

Several other risk factors have been identified. For example, gastrointestinal disorders with or without diarrhoea have been associated with a 2-fold increase in the risk of MDR Gram-negative acquisition [23]. Additionally, chronic intestinal disease has also been identified as a risk factor [3].

Other factors seem to influence the risk of attaining a MDR pathogen, such as the duration of stay and the presence of chronic disease [69,70]. Notably, the type of travel has also been identified as relevant. For example, travellers visiting family and friends face a higher risk of colonization, especially if sharing home-cooked food under suboptimal hygiene conditions. Those staying in private accommodations are also more likely to attain MDR strains when compared to those staying in tourist accommodation [62,71,72]. Staying in a hotel, as opposed to hostels or camping, seems to increase the risk as well [62]. Possible explanations include potential contamination during food preparation (more fresh food in hotels, cross-contamination in buffets, or greater trust in hotels leading to neglecting handwashing) [62,73]. Other studies present all-inclusive resorts as bearing a lower risk [22,69], while consuming street food, local ice creams, or local pastries have also been identified as risk factors [3,69]. Handwashing before meals, however, has been shown to be an important protective factor [3]. The association between age and the risk of colonization is inconsistent, with some studies indicating that younger travellers are at greater risk, while others report a higher risk among older travellers [23,24,67,74].

Another common risk factor for the acquisition of MDR Gram-negative bacteria is the use of antibiotics during travel, with an estimated 3-fold increase in risk [75,86]. Antibiotic residues can alter the composition of the intestinal microbiota by disturbing its balance and decreasing colonization resistance, and by promoting the overgrowth of resistant bacteria. In particular, beta-lactams and fluoroquinolones have been specifically identified as risk factors. Surprisingly, doxycycline, widely used as a malaria prophylaxis, was not associated with an increased risk of colonization [3,22], perhaps due to a lesser impact on the intestinal microbiome.

However, a synergistic effect seems to exist between the various risk factors [40]. In one study, the rate of MDR Gram-negative acquisition in individuals travelling to the Indian subcontinent was 23% in those who did not develop diarrhoea, 47% in those who had diarrhoea without antibiotic use, and 80% in those who had diarrhoea with antibiotic use [67]. A recent meta-analysis showed a 72% rate of ESBL-producing *E. coli* colonization among travellers to India who experienced traveller’s diarrhoea [68].

An often-underrated risk factor for acquiring MDR strains is direct contact with animals. Livestock, domestic pets, and wildlife can harbour resistant bacteria in their gastrointestinal systems or on their skin, acting as reservoirs for MDR pathogens [76,77]. In a Chinese study, 83% of *E. coli* isolates from primates were resistant to at least one antibiotic, and 48% exhibited multiple drug resistances [78]. The widespread use of antibiotics in agriculture, aquaculture, and veterinary care can expose bacteria to sub-inhibitory concentrations of these drugs, further promoting resistance [17,18,19].

Direct contact with animals, such as petting, handling, or consuming undercooked animal products, can facilitate the transmission of MDR bacteria to humans. Petting zoos, which allow both direct and indirect exposure to a wide range of animals, are particularly concerning. These settings have been linked to the shedding of Enterobacterales, including strains producing ESBL and AmpC beta-lactamases [77].

In a Finnish study including patients directly transferred from hospitals abroad, geographical region, ICU treatment, and antibiotic use abroad were identified as independent risk factors for MDR bacteria colonization [2].

## 7. Mass Gatherings as High-Risk Environments

Mass gatherings, such as the annual Hajj pilgrimage and the year-round Umrah rites in Saudi Arabia, which collectively attract around 10 million pilgrims annually from more than 180 countries [79], pose significant public health challenges, particularly regarding the spread of infectious diseases. While risks such as water- and sanitation-related disorders, non-communicable diseases, and non-infectious complications—like exacerbations of comorbidities, heat-related illnesses, trauma from stampedes or accidents, and substance abuse—are well recognized, an emerging and critical concern is the risk of colonization by MDR Gram-negative bacteria among attendees [79].

Mass gatherings present a unique risk for the transmission of MDR pathogens, as large numbers of individuals from diverse geographic regions—each with varying levels of healthcare infrastructure and antibiotic usage—converge in crowded, confined spaces. These conditions facilitate the spread of resistant bacteria, which can lead to outbreaks with widespread public health consequences. Additionally, the use of communal facilities, such as sleeping quarters, restrooms, dining areas, and shared transportation, further accelerates the transmission of infections [79].

A notable example of such an outbreak occurred at the 23rd World Scout Jamboree in Japan in 2015, where over 33,000 scouts from 162 countries attended. Following the event, imported cases of *Neisseria meningitidis* capsular group W were reported in European countries [80]. Among 1020 Swedish participants, 8% tested positive for *N. meningitidis* colonization, 2% of them with groupable strains, with half of these cases being of group W [81].

The overuse of antibiotics and their easy availability over the counter in many countries contribute significantly to the rise of MDR strains [16]. In the context of Hajj, there is concern regarding the spread of *N. meningitidis* from visitors arriving from the African meningitis belt. To mitigate this risk, a single dose of the ACWY135 meningococcal vaccine and a dose of ciprofloxacin have been recommended. However, this increases antibiotic exposure, contributing to selective pressure that promotes resistance [16]. A systematic review of 31 studies found a high prevalence of resistance to cephalosporins and carbapenems among Gram-negative bacteria, further emphasizing the risk posed by antibiotic resistance at mass gatherings [82].

In the case of Hajj, there is documented evidence of an increase in *E. coli* carriage between pre- and post-Hajj cohorts, with a particularly high prevalence of the *bla*CTX-M-15 gene. The widespread nature of ESBL-producing genes, their ease of transmission, and the crowded conditions during Hajj create an environment in which resistant bacteria are readily acquired and spread. This poses particular risks to pilgrims from countries with lower national prevalence of MDR organisms, who may encounter these resistant pathogens for the first time [83].

Similarly, the Kumbh Mela, one of the largest religious gatherings in the world, which takes place periodically in India, presents its own set of public health challenges [84]. During the event, millions of pilgrims engage in frequent and prolonged bathing in the holy rivers, leading to contamination of the water with bodily secretions. This practice facilitates the transmission of waterborne and respiratory pathogens [79]. Despite the availability of toilets during the 2013 and 2016 Kumbh Mela ceremonies, pilgrims were observed to engage in open defecation and urination, resulting in a dramatic increase in bacterial load in the river—up to 130-fold, with an ensuing increase in diarrhoeal diseases [84].

The widespread prevalence of MDR Gram-negative bacteria in India raises concerns about the spread of resistant strains during such events [3]. For example, a 20-fold increase in the presence of *bla*NDM-1 gene-carrying bacteria in the Upper Ganges River has been directly linked to seasonal pilgrimages, underscoring the risks associated with large-scale religious events [85]. The transmission of these resistant pathogens poses a threat not only to immediate attendees but also to global public health, as pilgrims return home with the potential to spread these bacteria to other parts of the world.

Other large-scale events, including major sporting events, music festivals, and religious gatherings, similarly carry the risk of MDR bacterial transmission. These events often share the same risk factors—crowded environments, communal facilities, and international travel—that facilitate the spread of infectious agents [79].

Given the global mobility of people and the rising prevalence of AMR, the vulnerability of travellers at mass gatherings, particularly in settings where healthcare resources may be limited and where the rapid onset of infectious outbreaks can overwhelm local healthcare systems, it is crucial to adopt a coordinated approach to mitigate the risks of MDR. Public health authorities, event organizers, and healthcare providers should collaborate to develop comprehensive strategies that address these emerging threats and safeguard the health of attendees and the global population.

## 8. Duration of Colonization and Overall Impact in Healthcare

There is no doubt that AMR is directly associated with an increased risk of severe infections with potentially high morbidity and mortality, the use of broader-spectrum antibiotics, increased selective pressure contributing to resistance, higher healthcare costs, and longer hospital stays [13,14,87,88,89]. Indeed, travel itself constitutes a risk factor for infection with MDR microorganisms. This has been particularly evident in community-acquired urinary tract infections caused by ESBL-producing Enterobacterales [90,91]. It has been reported that after travelling to Asia, Africa, or the Middle East, the risk of acquiring these infections is increased 21-fold (4.5–97) in the six weeks following return, 2.3-fold (1.2–4.4) between six weeks and one year after return, and is not significantly higher for trips taken between one and five years post-return [90].

Thus, colonization by MDR Gram-negative bacteria can be persistent. While colonization clearance occurs over time, with a median duration of less than 30 days, it appears to be slower in travellers returning from Asia compared to other regions. This likely reflects a higher intestinal bacterial load of MDR organisms, resulting from contact with a greater bacterial inoculum in Asian countries [3,22]. Nevertheless, overall, some authors have described carriage to persist in approximately 10% at a year post-travel [3]. Dall et al. reported 93.2% MDR Gram-negative acquisition rate after a trip to India, with 30.6% still being colonized after six months [92].

The acquisition of MDR strains in the context of travel and their potential for dissemination upon return is, therefore, concerning. Transmission of MDR strains within the household has already been documented [63,69], with the risk of transmission reaching 12% among colonized travellers [3]. Considering the epidemiology of tourism and migration, with billions of trips annually, many of which are to regions with a high risk of MDR strain acquisition, without impactful antimicrobial stewardship programmes and often a lack of effective public hygiene, this seemingly modest risk of secondary colonization takes on significant proportions. This is especially relevant when considering contact with vulnerable family members [66].

## 9. Prevention and Control of MDR Gram-Negative Bacteria Colonization

Globally, in regions lacking reliable access to clean water, sanitation, and hygiene, investment is crucial to prevent the rise and spread of AMR and to ensure the availability of effective, high-quality antimicrobials. The responsible use of antimicrobials must be prioritized globally to reduce the risk of AMR development. Widespread surveillance across all nations is thus essential to monitor and combat this escalating threat [93].

Travellers have relatively little information and concern regarding the risk of colonization by MDR Gram-negative bacteria. While information on AMR is widely available, the recognition that travel itself could pose a risk for acquiring an MDR strain is infrequent [94].

Thus, preventing colonization and infection by MDR Gram-negative requires a multifaceted approach, particularly for travellers heading to regions with high AMR prevalence. Key preventive strategies include:

- Pre-travel consultation and vaccination: Travellers should receive appropriate consultation and vaccinations before visiting endemic regions [95]. Vaccines for typhoid fever, cholera (only in high-risk settings such as outbreaks), and hepatitis A are readily available. While pre-travel receipt of the injected typhoid vaccine and the oral cholera vaccine have been associated with a reduction in the risk of *E. coli* acquisition, further research is needed to assess the true impact of protection and its possible mechanism [72,96].
- Hygiene and sanitation practices: Travellers should be educated on proper hygiene, including frequent handwashing with soap and water, using hand sanitizers, and avoiding the consumption of raw or undercooked food and untreated water [93]. Safe water consumption is particularly important in high-risk regions, where waterborne pathogens can carry resistant strains. Travellers should avoid petting stray or zoo animals and, if they do, they should follow appropriate sanitation measures afterwards.
- Antibiotic stewardship: Travellers should avoid self-medication and only use antibiotics as prescribed by a healthcare professional [93]. Educating travellers on the risks of misusing antibiotics can help curb the development of resistance. Albeit optimized hygiene practices can reduce the risk of traveller’s diarrhoea, should it happen, antibiotics ought to be used judiciously. There is no role for antimicrobial prophylaxis (other than in very selective high-risk health-related situations) [97]. In light or moderate diarrhoea, taking only antidiarrheal drugs such as loperamide is acceptable, with antimicrobial use being recommended in cases of severe diarrhoea or symptoms of dysentery [97]. No association has been observed between the use of loperamide without antibiotics and acquisition of MDR Gram-negatives [98].
- Avoid unnecessary hospitalization: Hospitalization is associated with colonization with Gram-negative bacteria [93,99]. Travellers should avoid seeking medical care unless absolutely necessary. When seeking healthcare abroad, if possible, it might be helpful to ensure that the facility adheres to stringent infection control protocols.
- Post-travel screening: Currently there is no recommendation for screening for MDR colonization upon returning home after travelling [97]. However, it might be useful in selected cases, especially for vulnerable travellers who needed to seek medical care abroad or were visiting friends and family, especially in high-risk regions, and present with a suspected bacterial infection. Early detection of colonization can help prevent further spread and ensure that proper infection control measures are in place if an infection develops. Nevertheless, as part of Antimicrobial Stewardship programmes, a thorough epidemiologic history and screen for MDR upon arrival to a healthcare facility might be useful.

The human microbiome is an effective barrier to infection, standing to reason that its preservation might be critical in preventing colonization by strains that are less well-adapted to the gut environment. Indeed, prior colonization with *Citrobacter freundii* and certain species of *Bacteroides* appear to be protective against the acquisition of MDR strains [100]. Consequently, the use of pre-/probiotics for the prevention or treatment of traveller’s diarrhoea and prevention of colonization with MDR strains is of interest due to their safety and ease of use. However, the evidence regarding their effectiveness is contradictory, and their use is generally not recommended [92,97,101,102].

## 10. Conclusions

In conclusion, the global movement of people has led to an increased risk of colonization with multidrug-resistant bacteria. Travellers and migrants coming from regions with high rates of antimicrobial resistance are at greater risk of acquiring MDR strains through various routes. Understanding the mechanisms of resistance, the risk factors for colonization, and the preventive strategies available is crucial for reducing the spread of these dangerous pathogens. Preventive measures, including pre-travel consultations, proper hygiene practices, and antibiotic stewardship, are essential for mitigating the risks associated with MDR colonization among travellers and migrants. However, there are still several gaps in the literature. Further investigation is warranted to clarify the true role, if any, of probiotics in the prevention of the acquisition of MDR strains. As the global burden of antimicrobial resistance continues to grow, international collaboration and strengthened infection control practices and public health policies are vital for curbing the spread of MDR bacteria and protecting public health.

## Figures and Tables

**Table 1:** Risk factors for MDR Gram-negative colonization in travellers and migrants.

Risk Factor	Reference
Travel to a low- or middle-income tropical or subtropical countries	Kajova et al. (2021) [2], Arcilla et al. (2017) [3], Tham et al. (2010) [4], Seijas-Pereda et al. (2024) [40], Meurs et al. (2020) [62], Voor In’T Holt et al. (2020) [63], Lübbert et al. (2015) [64], Schaumburg et al. (2019) [65], Ruppé et al. (2018) [66]
Travel to Asia—especially the Indian subcontinent	Tham et al. (2010) [4], Seijas-Pereda et al. (2024) [40], Meurs et al. (2020) [62], Voor In’T Holt et al. (2020) [63], Ruppé et al. (2018) [66]
Traveller’s diarrhoea	Östholm-Balkhed et al. (2013) [23], Kantele et al. (2015) [67], Muzembo et al. (2022) [68]
Chronic intestinal disease	Arcilla et al. (2017) [3]
Duration of stay	Kuenzli et al. (2014) [69], Vading et al. (2016) [70]
Visiting friends and family	Meurs et al. (2020) [62], Hassing et al. (2015) [71], Worby et al. (2023) [72]
Staying at a hotel	Meurs et al. [62], von Wintersdorff et al. (2014) [73]
Consuming street food, local ice creams, or local pastries	Arcilla et al. (2017) [3], Kuenzli et al. (2014) [69]
Age	Östholm-Balkhed et al. (2013) [23], Miranda et al. (2016) [24], Lääveri et al. (2018) [74], Kantele et al. (2015) [67]
Antibiotic use	Kajova et al. [2], Dethlefsen et al. (2008) [75], Kantele et al. (2015) [67]
Contact with animals	Ahmed et al. (2007) [76], Shnaiderman-Torban et al. (2019) [77], Zhu et al. (2021) [78]
Use of a healthcare facility abroad	Kajova et al. [2], Vading et al. (2016) [70]
Trip to mass gatherings	Memish et al. (2019) [79], Smith-Palmer et al. (2016) [80], Jacobsson et al. (2018) [81], Al-Tawfiq et al. (2015) [16], Leangapichart et al. (2017) [82], Pao et al. (2024) [83], Jani et al. (2018) [84], Ahammad et al. (2014) [85]
